# Verasense sensor-assisted total knee arthroplasty showed no difference in range of motion, reoperation rate or functional outcomes when compared to manually balanced total knee arthroplasty: a systematic review

**DOI:** 10.1007/s00167-023-07352-9

**Published:** 2023-03-01

**Authors:** Manuel-Paul Sava, Hitomi Hara, Leica Alexandra, Rolf W. Hügli, Michael T. Hirschmann

**Affiliations:** 1 Department of Orthopaedic Surgery and Traumatology, Kantonsspital Baselland (Bruderholz, Liestal, Laufen), CH-4101 Bruderholz, Switzerland; 2 Department of Clinical Research, Research Group Michael T. Hirschmann, Regenerative Medicine and Biomechanics, University of Basel, CH-4001 Basel, Switzerland; 3 Department of Orthopaedic Surgery, Kobe University Graduate School of Medicine, 7-5-1 Kusunoki-cho, Chuo-ku, Kobe, 650-0017 Japan; 4 Institute of Radiology and Nuclear Medicine, Kantonsspital Baselland (Bruderholz, Liestal, Laufen), CH-4101 Bruderholz, Switzerland

**Keywords:** Sensor assisted, Total knee arthroplasty, Total knee replacement, Verasense, Clinical outcomes, Functional outcomes, Intraoperative sensors, Systematic review

## Abstract

**Purpose:**

The aim of this systematic review was to investigate the clinical and functional knee outcomes after Verasense sensor-assisted total knee arthroplasty (VA TKA), and to compare these outcomes, where possible, with those from manually balanced total knee arthroplasty (MB TKA).

**Methods:**

A systematic literature search following PRISMA guidelines was conducted on PubMed, Embase, Medline and Scopus from the beginning of January 2012 until the end of June 2022, to identify potentially relevant articles for this review. Selection was based on the following inclusion criteria: full text English- or German-language clinical studies, published in peer-reviewed journals, which assessed clinical and functional outcomes following VA TKA. Not original research, preprints, abstract-only papers, protocols, reviews, expert opinion papers, book chapters, surgical technique papers, and studies pertaining only to unicondylar knee arthroplasty (UKA) or patellofemoral arthroplasty (PFA) were excluded. Several scores (Knee Society Score [KSS], Oxford Knee Score [OKS], Western Ontario and McMaster Universities Osteoarthritis Index [WOMAC], Knee injury and Osteoarthritis Outcome Score—4 subscales [KOOS4] and Physical Function—Computerised Adaptive Testing [PF˗CAT]), alongside postoperative measurements of range of motion [ROM], reoperation rates and the rate of manipulation under anaesthesia [MUA]) were used to evaluate clinical and functional outcomes. The quality of included papers, except randomised control trials (RCTs), was evaluated using the Methodological Index for Non-Randomised Studies (MINORS). For the assessment of included RCTs, the Jadad Scale was used.

**Results:**

The literature search identified 243 articles. After removing duplicates, 184 papers were included in the initial screening process. Fourteen of them met all the inclusion criteria following the selection process. Mean MINORS for non-comparative studies value was 11.5 (11–12), and for comparative studies 18.2 (13–21). Mean Jadad Scale score was 3.6 (2–5). Outcomes from a total number of 3633 patients were evaluated (mean age at surgery 68.5 years [32–88 years]). In terms of clinical outcomes, the overwhelming majority of studies observed an improvement after VA TKA, but no statistically significant difference in ROM and reoperation rate when compared to MB TKA. On the other hand, lower rates of MUA have been described in the VA TKA group. An increase in postoperative clinical and functional scores values, when compared to the preoperative ones, has been reported in both groups, although no statistically significant difference between them has been observed.

**Conclusion:**

The use of Verasense pressure sensors in TKA leads to no significant improvement in ROM, reoperation rate or functional outcomes, when compared to the standard manually balancing technique. However, lower rates of MUA have been described in the VA TKA group. These findings highlight the importance of tools being able to measure ligament stresses or joint pressure for achieving an optimally balanced knee.

**Level of evidence:**

III.

**Supplementary Information:**

The online version contains supplementary material available at 10.1007/s00167-023-07352-9.

## Introduction

Achieving optimal clinical and functional outcomes after total knee arthroplasty (TKA) depends on a number of factors such as leg alignment, orientation of the components, as well as adequate soft tissue balancing [2, 9, 21, 24]. Assessing soft tissue balance is challenging for the surgeon, as the perceived “feeling for balance” is influenced by several elements such as surgical experience, patients’ body mass index (BMI), gender, soft tissue laxity, the degree of joint contracture, as well as the fitness level of the surgeon [14]. Consequently, this might lead to an unbalanced knee as well as further revisions, which both reduce the patients’ quality of life [11]. Hence, the development of new methods to assess intraoperative ligament laxity and balancing is important [28]. One of the current debated methods is the use of intraoperative sensor technology, which allows surgeons to dynamically quantify in vivo joint laxity and perform a more objective soft tissue balancing [26]. These sensors enable the surgeon to receive objective intraoperative digital feedback [17]. Intraoperative pressure sensors may play an important role in reducing dissatisfaction related to knee stiffness or instability [11]. Despite their ability to improve soft tissue balance, it is not clear whether the use of intraoperative sensors improve clinical and functional outcomes in TKA. Several studies have shown improved early outcomes with the use of sensors [8, 11, 12], whereas others have failed to demonstrate any clinical benefit [18, 25]. Although an optimal balance of the medial and lateral joint gaps in flexion and extension has been considered an important determinant of clinical and functional outcomes, improved outcomes when compared with TKAs that are not balanced, have not yet been proven [9, 17]. One of the commercially available intraoperative sensors is the Verasense sensor [17]. Due to its widespread use, and to the fact that the majority of the studies, which report on clinical and functional outcomes following sensor-assisted TKA, are using Verasense, the present study focuses on its use. The aim of this systematic review was to investigate the clinical and functional knee outcomes after Verasense sensor-assisted TKA (VA TKA) and to compare these outcomes where possible with those from manually balanced TKA (MB TKA). The hypothesis was VA TKA does yield satisfactory postoperative clinical and functional outcomes, but not superior to those after MB TKA.

## Materials and methods

A systematic literature search was conducted on PubMed, Embase, Medline and Scopus to identify potentially relevant articles published between June 2012 and June 2022. Terms including “intraoperative sensor”, “sensor technology”, “sensor assisted”, “Verasense”, “pressure sensor”, “force sensor”, “sensor balancing”, “total knee arthroplasty”, “knee revision”, “knee replacement”, “revision arthroplasty”, “TKA” and “TKR” were searched for in both title and abstract. Detailed information regarding the used search strategy can be found in “Additional file 1”.

Inclusion criteria were full text English- or German-language clinical studies, published in peer-reviewed journals that assessed the clinical and functional outcomes following VA TKA. Only original research articles were considered. Preprints, abstract-only studies, protocols, reviews, expert opinion studies, book chapters, surgical technique studies, and studies pertaining only to unicondylar knee arthroplasty (UKA) or patellofemoral arthroplasty (PFA), were excluded. Only articles reporting clinical and functional knee outcomes after VA TKA were included.

After collecting all articles and removing duplicates, two authors screened the studies by title and abstract analysis for inclusion. In a second step, selected articles were checked for their eligibility by full text analysis. In case of uncertainty regarding inclusion a third author was consulted. A further manual screening of the reference lists of included articles was done for additional studies. For this review, only studies reporting clinical and functional outcomes were included. Endpoints included various postoperative patient-reported outcome measures (PROMs) such as Knee Society Score (KSS), Oxford Knee Score (OKS), Knee Injury and Osteoarthritis Outcome Score—4 Subscales (KOOS4), Western Ontario and McMaster Universities Osteoarthritis Index (WOMAC) and Physical Function—Computerised Adaptive Testing (PF˗CAT), as well as range of motion (ROM), rate of arthrofibrosis and reoperation rate.

### Quality assessment

The methodological quality of the included comparative and non-comparative non-randomised clinical studies was assessed by two raters independently, using the Methodological Index for Non-Randomised Studies (MINORS). The global ideal score is 16 for non-comparative studies, and 24 for comparative studies. For the assessment of included randomised control trials (RCTs), the Jadad Scale was used, with the maximum score being 5. The level of evidence of the included studies was also reported.

### Data extraction

From the selected publications, title, author, year of publication, study design, level of evidence, number of knees, follow-up time, patient demographics, clinical outcome scores and functional outcomes were extracted.

### Statistical analysis

Continuous variables were described with mean and standard deviation or median and range. Categorical variables were reported with absolute and relative frequencies. A *p* < 0.05 was considered statistically significant.

## Results

### Search results and characteristics of included studies

The literature search identified 243 scientific articles in the initial screening. A total of 11 met all the inclusion criteria following the selection process shown in Fig. 1 (Additional file 2). Median MINORS for non-comparative studies value was 11.5 (11–12), and for comparative studies 18.2 (13–21). Median Jadad Scale score was 3.6 (2–5). Results from a total number of 3633 patients were evaluated (median age at surgery 68.5 years, range 32–88 years). The reported median body mass index (BMI) was 30.8 (17–45). Several scores (KSS, OKS, WOMAC, KOOS4, PF˗CAT), alongside postoperative ROM, reoperation rates and the rate of manipulation under anaesthesia (MUA), were used to evaluate clinical and functional outcomes. The detailed characteristics of the included studies are presented in Table 1 (Additional file 3).

### Clinical outcomes

ROM was evaluated in five studies (Table 1). They reported no statistically significant differences between the VA and MB groups [5, 7, 19, 25, 29]. Mean follow-up time ranged from 3 to 24 months.

**Table 1:** Summary of reported flexion

Author (year)	Groups (no.)	Follow-up time (mean ± SD)	Preoperative value (mean ± SD)	*p* value	Postoperative value (mean ± SD, %)	*p* value
Geller (2016**) **[7]	VA (292)	3 months	110°	< 0.001	1[12]°	n.s
	MB (690)		104.5°		113.2°	
Cochetti (2020) [5]	VA (50)	24 months	112° ± 11.8°	n.s	124° ± 11.5°	n.s
	MB (50)		108° ± 11.8°		123° ± 10.7°	
MacDessi (2022) [19]	VA (144)	6 months	nm		105.3° ± 12.9°	n.s
	MB (141)				105.3° ± 15.07°	
Song (2019) [25]	VA (50)	6 months	126.6° ± 16.2°	nm	132.6° ± 5.3°	nm
	MB (50)		122.7° ± 14.2°		131.8° ± 7.0°	
Wood (2020) [29]	VA (76)	12 months	109.3° ± 11.5°	n.s	118.2° ± 9.1°	n.s
	MB (76)		105.4° ± 13.2°		116.2° ± 11.8°	

*SD *standard deviation, *VA* Verasense sensor assisted, *MB* manually balanced, *nm *not mentioned

Nine studies examined the rate of MUA as an assessment of postoperative arthrofibrosis (Table 2). Geller et al. reported that the rate of MUA in the MB TKA group was higher than in the VA TKA group, and that the period between TKA and MUA was on average 13.5 weeks and 7.5 weeks, respectively [15]. Some of the other studies also showed higher rates of MUA in the MB TKA group, but with no significant differences. The mean follow-up time ranged from 6 to 24 months [1, 3, 5, 7, 16, 18, 19, 25, 29].

**Table 2:** Summary of reported rate of MUA

Author (year)	Groups (no.)	Follow-up time (mean ± SD)	Rate (no. of cases, %)	*p* value
Geller (2016) [7]	VA (292)	3 months	4 (1.40%)	0.004
	MB (690)		34 (5.00%)	
Amundsen (2017) [1]	VA (75)	12 months	0 (0.00%)	n.s
	MB (225)		8 (3.50%)	
Chow (2017) [3]	VA (57)	6 months	1 (1.80%)	n.s
	MB (57)		3 (5.30%)	
Cochetti (2020) [5]	VA (50)	24 months	1 (2.10%)	nm
	MB (50)		1 (2.10%)	
Livermore (2018) [16]	AN + MB (103)	12 months	2 (1.94%)	n.s
	AN + VA (74)		1 (1.40%)	n.s
MacDessi (2021) [18]	VA (222)	24 months	9 (3.60%)	n.s
	MB (207)		8 (4.30%)	
MacDessi (2022) [19]	VA (144)	6 months	3 (2.40%)	nm
	MB (141)		7 (4.80%)	
Song (2019) [25]	VA (50)	8.8 ± 2.0 months	0 (0.00%)	nm
	MB (50)		0 (0.00%)	
Wood (2020) [29]	VA (76)	12 months	2 (2.63%)	nm
	MB (76)		2 (2.63%)	

*SD* standard deviation, *VA* Verasense sensor assisted, *MB* manually balanced, *ROM* range of motion, *MUA* manipulation under anaesthesia, *nm* not mentioned, *IMG* intramedullary guided, *AN* accelerometer navigated

Livermore et al. and MacDessi et al. reported the reoperation rate at a mean follow-up of 12 months and 24 months, respectively, with no significant differences [16, 18].

### Patient-reported outcome measures

Four studies have evaluated pre- and postoperative KSS (Table 3). These studies showed improved postoperative KSS, for both groups (VA TKA and MB TKA), but with no significant differences between the analysed groups at 6 and 12 months [3, 5, 25].

**Table 3:** Summary of reported KSS scores

Author (year)	Groups	KSS type	Follow-up time (mean ± SD)	Preoperative score (mean ± SD)	*p* value	Postoperative score (mean ± SD)	*p* value
Chow (2017) [3]	VA (57)	KSS total	6 months	Δ63 (nm) Δ52 (nm)			< 0.001
	MB (57)						
Cochetti (2020) [5]	VA (50)	KSS total	24 months	64.4 ± 3.9	n.s	165.7 ± 7.3	n.s
	MB (50)			65 ± 4.0		166.3 ± 3.9	
Song (2019) [25]	VA (50)	KSS total	6 months	79.7 ± 9.2	nm	169.3 ± 5.9	nm
	MB (50)			78.7 ± 9.4		166.1 ± 6.1	
Wood (2020) [29]	VA (76)	KSS Clinical	12 months	49.7 ± 13.1	n.s	90.0 ± 11.9	n.s
	MB (76)			49.6 ± 17.4		92.0 ± 10.2	

*SD* standard deviation, *nm* not mentioned, Δ mean score change, *VA* Verasense sensor assisted, *MB* manually balanced, *KSS* Knee Society Score, *nm* not mentioned

Three studies evaluated pre- and postoperative OKS (Table 4). Although the 6-month OKS scores were higher in the VA TKA group (*p* = 0.01), no significant differences were found between the two groups at 1- and 2-year follow-ups [3, 5, 29].

**Table 4:** Summary of reported OKS scores

Author (year)	Groups	Follow-up time (mean ± SD)	Preoperative score (mean ± SD)	*p* value	Postoperative score (mean ± SD)	*p* value
Chow (2017) [3]	VA (57)	6 months	Δ17 (nm) Δ13 (nm)			0.025
	MB (57)					
Cochetti (2020) [5]	VA (50)	24 months	19.6 ± 2.9	n.s	41.5 ± 4.1	n.s
	MB (50)		19.0 ± 2.53		41.1 ± 4.0	
Wood (2020) [29]	VA (76)	12 months	37.9 ± 7.1	n.s	20.5 ± 5.7	n.s
	MB (76)		38.8 ± 7.6		19.0 ± 7.0	

*SD* standard deviation, *nm* not mentioned, Δ mean score change, *VA* Verasense sensor assisted, *MB* manually balanced, *nm* not mentioned, *OKS* Oxford Knee Score

Three studies have reported preoperative and 1- and 2-year postoperative KOOS and WOMAC after VA and MB TKA [16, 19, 25]. They described improved postoperative scores in both groups, but with no significant differences between them. Detailed information can be found in Table 5.

**Table 5:** Summary of other reported PROMs

Author (year)	Groups	Type	Follow-up time (mean ± SD)	Preoperative score (mean ± SD)	*p* value	Postoperative score (mean ± SD)	*p* value
MacDessi (2022) [19]	VA (144) MB (141)	WOMAC	24 months	54.5 ± 18.1	nm	14.0 ± 14.4	nm
				54.6 ± 15.7		13.0 ± 13.8	
		KOOS4		38.1 ± 15.0		82.7 ± 15.1	
				38.0 ± 14.3		82.1 ± 15.5	
MacDessi (2021) [18]	VA (222)	KOOS4	24 months	Δ41.5 ± 16.9			n.s
	MB (207)			Δ42.4 ± 19.4			
Song (2019) [25]	VA (50)	WOMAC	12 months	71.7 ± 2.9	nm	17.0 ± 2.9	nm
	MB (50)			69.3 ± 3.3		17.0 ± 1.8	
Livermore (2020) [16]	VA (74) MB (103)	KOOS4	12 months	51.7 ± 15.3	nm	78.2 ± 16.7	nm
				49.8 ± 11.5		76.8 ± 17.1	
		PF-CAT		nm		45.1 ± 8.1	
						46.6 ± 6.6	

*SD* standard deviation, *nm* not mentioned, Δ mean score change, *VA* Verasense sensor assisted, *MB* manually balanced, *WOMAC* Western Ontario and McMaster Universities Osteoarthritis Index, *KOOS4* Knee Injury and Osteoarthritis Outcome Score—4 subscales, *PROMIS nm* not mentioned, *PF-CAT* Physical Function—Computerised Adaptive Test

## Discussions

The main findings of this study are that both VA TKA and MB TKA showed improved clinical and functional scores, but that there is no statistically significant difference in between VA TKA and MB TKA [5, 7, 19, 25, 29]. Similar findings were shown for PROMs (OKS, KSS, WOMAC, KOOS4 and PF-CAT) [5, 16, 18, 19, 25, 29]. There is only one exception, which has been also reported by Chow et al. who described a statistically significant difference (*p* < 0.025) in OKS at 6-month follow-up between VA TKA and MB TKA groups [3]. The VA TKA group scored higher (Δ17 vs Δ13). A similar difference for the KSS (Δ63 vs. Δ52) has also been shown by Chow et al. [3]. However, the findings although significant do not represent clinically relevant differences. In addition, in this study, only the mean change between preoperative OKS/KSS and 6-month postoperative OKS/KSS and not the absolute values were presented.

The most interesting finding of the present review is the fact that when no pressure sensors were used for TKA, the knee was more likely to undergo a manual manipulation [1, 3, 7, 16, 17, 19, 29]. However, although there was clear difference for a higher need for MUA in the conventional groups, the findings were not always statistically significant. This might be due to the laxity differences in the included knees. Many studies have just recently highlighted the importance of the envelope of laxity [9, 10]. In line with the ongoing knee phenotype discussion, with regards to alignment parameters, the identification of different knee phenotypes for the individual envelope of laxity has just begun [20, 27]. When having identified different knee laxity types, the question arises how to measure these pre-, intra- and postoperatively. Pre- and postoperatively stress radiographs might play a major role. Intraoperative sensor technology, navigational or robotic devices might also be helpful. However, all technologies available are still in the early childhood and needs to mature to make a real clinical impact in TKA. The biggest obstacle besides availability is questionable inter- and intra-observer reliability of all such tools, when measuring stress or pressure in the different joint compartments.

The findings of this review are in line with the findings of Sun et al. in their metanalysis [26]. The number of included studies was lower than in ours. Since then, many more studies have been published. Another difference lies in the availability and the language of the studies included. In the present review, no articles published in other languages aside English and German were included. In addition, the articles had to possess identification numbers (i.e. PID, DOI), or these had to be accessible (e.g. in online medical libraries [i.e. PubMed, Scopus, Embase, Medline, Google Scholar] and/or peer-reviewed journals).

This study presents a number of limitations. The low number of RCTs analysed in our review (i.e. three) combined with the inferior quality of some non-randomised clinical studies limits the level of evidence. The low number of enrolled patients in the majority of analysed studies could also play a major role as the ability to identify a statistically significant difference between study groups is heavily linked to the sample size. Although all present completed studies pertaining to this subject have been included, more RCTs and prospective studies are currently in different stages of completion [4, 23]. Their results are yet to be reported; thus, there is a possibility their future findings might not concur with ours. The variability between included studies regarding surgical techniques, type of prosthesis (i.e. cruciate retaining [CR] or posterior stabilised [PS]), endpoints, outcomes, follow-up periods and size of cohorts adds to the heterogeneity of the study samples. In addition, the follow-up period for these studies is relatively short, with no study analysing long-term outcomes (clinical or functional). In the end, this systematic review only assessed studies which included a certain type of intraoperative sensors (Verasense). Several other sensors are available on the market (e.g. eLibra, Omnibot), to which our findings may not apply [6, 15]. The number of available studies reporting on outcomes following these sensors is low though, and there is none comparing those outcomes to the ones following MB TKA. Therefore, inclusion of these studies in the review, and a subsequent comprehensive analysis of their findings, has not been performed.

## Conclusion

The use of Verasense pressure sensors in TKA leads to no significant improvement in ROM, reoperation rate or functional outcomes, when compared to the standard manually balancing technique. However, lower rates of MUA have been described in the VA TKA group.

## Supplementary Information

Below is the link to the electronic supplementary material.

Supplementary file1 (DOCX 125 KB)

Supplementary file2 Flow-chart of the study selection process according to the PRISMA 2020 statement: an updated guideline for reporting systematic reviews [29]—available as Additional file 2 (DOCX 17 KB)

Supplementary file3 Overview of selected studies—available as Additional file 3 (DOCX 40 KB)

## Data Availability

Data is available on request in personal repository.

## Abbreviations

TKA: Total knee arthroplasty
BMI: Body mass index
VA: Verasense assisted
MB: Manually balanced
UKA: Unicondylar knee arthroplasty
PFA: Patellofemoral arthroplasty
PROMs: Patient-reported outcome measures
KSS: Knee Society Score
OKS: Oxford Knee Score
KOOS4: Knee Injury and Osteoarthritis Outcome Score—4 subscales
WOMAC: Western Ontario and McMaster Universities Arthritis Index
PF-CAT: Physical Function—Computerised Adaptive Test
ROM: Range of motion
MINORS: Methodological Index for Non-Randomised Studies
RCT: Randomised control trial
MUA: Manipulation under anaesthesia
CR: Cruciate retaining
PS: Posterior stabilised
