# Supplementary material for: Verasense sensor-assisted total knee arthroplasty showed no difference in range of motion, reoperation rate or functional outcomes when compared to manually balanced total knee arthroplasty: a systematic review
Source: Knee Surg Sports Traumatol Arthrosc. 2023 Mar 1;31(5):1851–8. doi: 10.1007/s00167-023-07352-9 (PMC10090011; doi:10.1007/s00167-023-07352-9)
Supplement: Supplementary file 1 — Supplementary file1 (DOCX 125 KB) [file 167_2023_7352_MOESM1_ESM.docx]

**Search Strategy**

**Research question:** What are the clinical and functional outcomes of **VERASENSE** sensor assisted **TKA?**

**Concept 1: Sensors/ VERANSENSE**

**Keywords: “**intraoperative sensor*” OR “intra**-**operative sensor*” OR “sensor technology” OR VERANSENSE OR “sensor based” OR “force sensor*” OR “sensor assisted” OR “pressure sensor*” OR sensor*

**Concept 2: TKA / Revision TKA**

**Keywords**: "total knee arthroplasty*"[tw] OR TKA[tw] OR "knee revision*"[tw] OR "revision arthroplasty*"[tw]

**MesH:** "Arthroplasty, Replacement, Knee"[Mesh]

**PUBMED -78 results**

(("intraoperative sensor*"[Text Word] OR "intra operative sensor*"[Text Word] OR "sensor technology"[Text Word] OR "VERASENSE"[Text Word] OR "force sensor*"[Text Word] OR "sensor assisted"[Text Word] OR "pressure sensor*"[Text Word] OR "sensor*"[Text Word] OR "sensor balancing*"[Text Word]) AND ("2012/06/23 00:00":"3000/01/01 05:00"[Date - Publication] AND "loattrfull text"[Filter]) AND (("arthroplasty, replacement, knee"[MeSH Terms] OR "total knee arthroplasty*"[Text Word] OR "TKA"[Text Word] OR "knee revision*"[Text Word] OR "revision arthroplasty*"[Text Word]) AND ("2012/06/23 00:00":"3000/01/01 05:00"[Date - Publication] AND "loattrfull text"[Filter]))) AND ((y_10[Filter]) AND (clinicalstudy[Filter] OR clinicaltrial[Filter] OR controlledclinicaltrial[Filter] OR randomizedcontrolledtrial[Filter]) AND (fft[Filter]))

**EMBASE and MEDLINE - 102 results**

('total knee arthroplasty'/exp OR 'total knee arthroplasty' OR tka OR 'revision arthroplasty'/exp OR 'revision arthroplasty' OR 'knee replacement'/exp OR 'knee replacement') AND ('sensor' OR 'pressure sensor' OR 'verasense' OR 'force sensor' OR 'intraoperative sensor') AND [humans]/lim AND [english]/lim AND [abstracts]/lim AND [clinical study]/lim AND ([english]/lim OR [german]/lim) AND [2012-2022]/py AND ([embase]/lim OR [medline]/lim) AND ('case control study'/de OR 'case report'/de OR 'clinical article'/de OR 'clinical study'/de OR 'clinical trial'/de OR 'cohort analysis'/de OR 'comparative study'/de OR 'controlled clinical trial'/de OR 'controlled study'/de OR 'cross sectional study'/de OR 'major clinical study'/de OR 'multicenter study'/de OR 'observational study'/de OR 'prospective study'/de OR 'randomized controlled trial'/de OR 'retrospective study'/de) AND 'article'/it

**Scopus – 63 results**

TITLE-ABS-KEY(("total knee arthroplasty*" OR TKA OR "knee revision*" OR "revision arthroplast*") AND ("intraoperative sensor*" OR VERASENSE OR "force sensor*" OR "sensor assist*" OR "pressure sensor*" OR "sensor balanc*")) AND ( LIMIT-TO ( DOCTYPE,"ar" ) ) AND ( LIMIT-TO ( SUBJAREA,"MEDI" ) ) AND ( LIMIT-TO ( PUBYEAR,2022) OR LIMIT-TO ( PUBYEAR,2021) OR LIMIT-TO ( PUBYEAR,2020) OR LIMIT-TO ( PUBYEAR,2019) OR LIMIT-TO ( PUBYEAR,2018) OR LIMIT-TO ( PUBYEAR,2017) OR LIMIT-TO ( PUBYEAR,2016) OR LIMIT-TO ( PUBYEAR,2015) OR LIMIT-TO ( PUBYEAR,2014) OR LIMIT-TO ( PUBYEAR,2013) OR LIMIT-TO ( PUBYEAR,2012) )
