# Supplementary material for: Verasense sensor-assisted total knee arthroplasty showed no difference in range of motion, reoperation rate or functional outcomes when compared to manually balanced total knee arthroplasty: a systematic review
Source: Knee Surg Sports Traumatol Arthrosc. 2023 Mar 1;31(5):1851–8. doi: 10.1007/s00167-023-07352-9 (PMC10090011; doi:10.1007/s00167-023-07352-9)
Supplement: Supplementary file 2 — Supplementary file2 Flow-chart of the study selection process according to the PRISMA 2020 statement: an updated guideline for reporting systematic reviews [29]—available as Additional file 2 (DOCX 17 KB) [file 167_2023_7352_MOESM2_ESM.docx]

**Identification of studies via databases and registers**

Records removed *before screening*:

Duplicate records removed

(n = 59)

Records marked as ineligible by automation tools (n = 0)

Records removed for other reasons (n = 0)

Records identified from:

Pubmed (n = 78)

Scopus (n = 63)

Embase and Medline (n = 102)

**Identification**

Records excluded

(n = 152)

Records screened

(n = 184)

Reports not retrieved

(n = 2)

Reports sought for retrieval

(n = 32)

**Screening**

Reports excluded:

Wrong outcomes (n = 9)

Other intraoperative sensors (n = 2)

Wrong study design (n = 2)

Abstract-only paper (n = 2)

Expert opinion paper (n = 1)

Surgical technique paper

(n = 1)

Wearable sensor (n = 1)

Non-numerical data (n = 1)

Reports assessed for eligibility

(n = 30)

Studies included in review

(n = 11)

**Included**
