# Supplementary material for: Verasense sensor-assisted total knee arthroplasty showed no difference in range of motion, reoperation rate or functional outcomes when compared to manually balanced total knee arthroplasty: a systematic review
Source: Knee Surg Sports Traumatol Arthrosc. 2023 Mar 1;31(5):1851–8. doi: 10.1007/s00167-023-07352-9 (PMC10090011; doi:10.1007/s00167-023-07352-9)
Supplement: Supplementary file 3 — Supplementary file3 Overview of selected studies—available as Additional file 3 (DOCX 40 KB) [file 167_2023_7352_MOESM3_ESM.docx]

**Title: Verasense sensor assisted total knee arthroplasty leads to no difference in range of motion, reoperation rate or functional outcomes when compared to manually balanced total knee arthroplasty: a systematic review**

**Journal: Knee Surgery, Sports traumatology, Arthroscopy (KSSTA)**

**Authors: *Manuel-Paul Sava, Hitomi Hara, Leica Alexandra, Rolf W. Huegli, Michael T. Hirschmann***

Corresponding Author**: Prof. Michael T. Hirschmann**

- Head of the Department of Orthopaedic Surgery and Traumatology, Kantonsspital Baselland (Bruderholz, Liestal, Laufen), CH-4101 Bruderholz, Switzerland;
- Head of the Department of Clinical Research, Research Group Michael T. Hirschmann, Regenerative Medicine & Biomechanics, University of Basel, CH-4001 Basel, Switzerland;

Email: [michael.hirschmann@unibas.ch](mailto:michael.hirschmann@unibas.ch);

Tel.: +41-765105865

**Table 1.** Overview of selected studies

| **Author (year)** | **Number of knees** | **Study type** | **Mean age, years (SD, range)** | **Gender male (%)** | **Mean BMI (SD, range)** | **Mean follow-up time (SD, range)** | **Level of evidence** | **MINORS/ Jadad Score** |
| --- | --- | --- | --- | --- | --- | --- | --- | --- |
| Chow (2017) [10] | 114 knees  (114 patients) | Retrospective cohort | 66.3 (nm)^a^ | 43.8%^a^ | 29.5 (nm) ^a^ | 6 months (nm) | III | 19/24 |
| Cochetti (2020) [14] | 100 knees  (100 patients) | Prospective matched-pair case-control | 67.5 (nm, 48-77)^a^ | 96%^a^ | 34.4 (nm, 22-42.5) ^a^ | 24 months (nm) | III | 21/24 |
| Geller (2016) [15] | 942 knees  (942 patients) | Retrospective cohort | 68 (±9.7, nm)^a^ | 23%^a^ | 31.5 (nm) ^a^ | 3 months (nm) | III | 13/24 |
| Gustke (2014) [9] | 176 knees  (176 patients) | Prospective cohort | 70.5 (±7.5, nm)^a^ | 33.3%^a^ | 30.5 (±5.6, nm) ^a^ | 6 months (nm) | II | 11/16 |
| Gustke (2014) [16] | 135 knees  (135 patients) | Prospective cohort | 70.5 (±7.5, nm)^a^ | 33.3%^a^ | 30.5 (±5.6, nm) ^a^ | 12 months (nm) | II | 12/16 |
| Livermore (2018) [17] | 371 knees  (371 patients) | Retrospective cohort | 67 (±8.8, 32-88)^a^ | 55.5%^a^ | 30 (±5.7, 17-45) ^a^ | 12 months (nm) | III | 18/24 |
| MacDessi (2021) [13] | 429 knees  (375 patients) | Retrospective cohort | 67.3 (nm)^a^ | 62.2%^a^ | 29.9 (nm) ^a^ | 24 months (nm) | III | 20/24 |
| MacDessi (2022) [18] | 285 knees  (250 patients) | RCT | 69.1 (±8.4, nm)^a^ | 56%^a^ | 31.6 (±6.5, nm) ^a^ | 24 months (nm) | I | 4/5 |
| Song (2019) [12] | 100 knees  (100 patients) | RCT | 72.6 (±6.3, nm)^a^ | 15%^a^ | 26.2  (±3.4, nm)^a^ | 8.6 months (±2.1, nm) | I | 2/5 |
| Wood (2020) [19] | 152 knees  (152 patients) | RCT | 66.9 (±7.5, nm)^a^ | 34.5%^a^ | 33.5  (±6, nm)^a^ | 12 months (nm) | I | 5/5 |
| Amundsen (2017) [20] | 300 knees  (302 patients) | Retrospective cohort | 68.5 (±8.6, nm)^a^ | 31.2%^a^ | 31.7  (±5.8, nm)^a^ | 12 months (nm) | III | 20/24 |

Abbreviations: BMI: body mass index (kilogram/meter^2^), SD: standard deviation, nm: not mentioned, RCT: randomized control trial.

^a^ Values from multiple groups combined into one overall group
